# Targeted Next-Generation Sequencing Revealed Novel Mutations in Chinese Ataxia Telangiectasia Patients: A Precision Medicine Perspective

**DOI:** 10.1371/journal.pone.0139738

**Published:** 2015-10-06

**Authors:** Zhao Chen, Wei Ye, Zhe Long, Dongxue Ding, Huirong Peng, Xuan Hou, Rong Qiu, Kun Xia, Beisha Tang, Hong Jiang

**Affiliations:** 1 Department of Neurology, Xiangya Hospital, Central South University, Changsha, Hunan, P.R. China; 2 Key Laboratory of Hunan Province in Neurodegenerative Disorders, Central South University, Changsha, Hunan, P.R. China; 3 State Key Laboratory of Medical Genetics, Central South University, Changsha, Hunan, P.R. China; 4 School of Information Science and Engineering, Central South University, Changsha, Hunan, P.R. China; Cornell University, UNITED STATES

## Abstract

Ataxia telangiectasia (AT) is an autosomal recessive disease characterized by progressive cerebellar ataxia, oculocutaneous telangiectasia and immunodeficiency due to mutations in the *ATM* gene. We performed targeted next-generation sequencing (NGS) on three unrelated patients and identified five disease-causing variants in three probands, including two pairs of heterozygous variants (FAT–1:c.4396C>T/p.R1466X, c.1608-2A>G; FAT–2:c.4412_4413insT/p.L1472Ffs*19, c.8824C>T/p.Q2942X) and one pair of homozygous variants (FAT–3: c.8110T>G/p.C2704G, Hom). With regard to precision medicine for rare genetic diseases, targeted NGS currently enables the rapid and cost-effective identification of causative mutations and is an updated molecular diagnostic tool that merits further optimization. This high-throughput data-based strategy would propel the development of precision diagnostic methods and establish a foundation for precision medicine.

## Introduction

Ataxia telangiectasia (AT, MIM#208900), due to mutations in the ataxia telangiectasia mutated gene (*ATM*, MIM*600118), is an autosomal recessive disease characterized by progressive cerebellar ataxia, oculocutaneous telangiectasia and immunodeficiency, as well as elevated α-fetoprotein (AFP) serum levels, immunoglobulin deficiency and predisposition to cancers[1–4]. Typically occurring early in childhood, AT patients usually die in their twenties due to malignancies or respiratory failure. Since two Chinese AT patients were first described in our previous work, very few AT cases have been reported in China [5–7]. For such a rare genetic disease, the advent of precision medicine that aims to generate individualized approaches for prevention, diagnosis and treatment would provide broad insight into genetic diagnosis and counselling [8]. Targeted next-generation sequencing (NGS) currently allows the rapid and cost-effective identification of causative mutations and is an updated molecular diagnostic tool that merits further optimization. In this study, we performed targeted NGS on three unrelated AT patients whose diagnoses were confirmed by the identification of disease-causing variants, thereby illustrating the utility of NGS in precision medicine.

## Patients and Methods

### Patients

**Fig 1:**
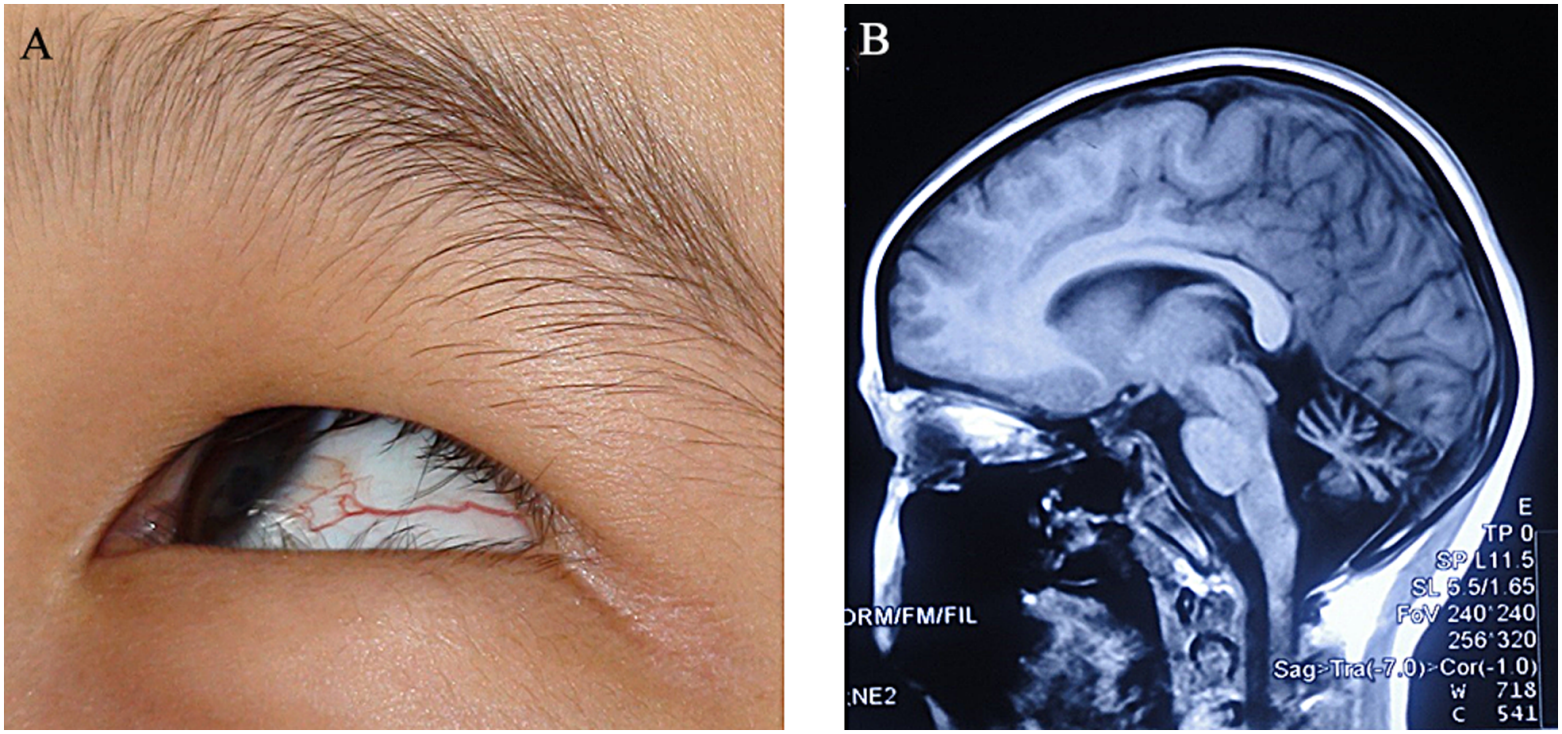
The clinical features of an AT patient. Ocular telangiectasia (A) and cerebellar atrophy revealed on brain MRI (sagittal T1) (B) were indicated in a proband (FAT–2 pedigree) with AT.

Three unrelated patients (two males, one female in FAT–1, FAT–2 and FAT–3 families respectively) suspected of having AT were recruited in the study. These patients underwent clinical investigations including neurologic examinations, laboratory tests and brain MRI evaluations. The primary clinical diagnosis for each individual was mainly based on the symptoms of progressive cerebellar ataxia, dysarthria and oculocutaneous telangiectasia, as well as signs of cerebellar atrophy revealed on MRI, elevated serum α-fetoprotein, and altered immunoglobulin profiles (Fig 1, Table 1). Meanwhile, their parents were clinically unaffected without any neurological involvement.

**Table 1:** Clinical and laboratory features of each AT individual.

Family	Gender	Age (year)	Age at onset (year)	Presenting feature	SARA(score)	ICARS(score)	Bain MRI	α-fetoprotein(ng/ml)^a^	Immunoglobulins		
									lgG(g/L)^b^	lgA(g/L)^c^	lgM(g/L)^d^
FAT–1	male	7	2	ataxia, oculocutaneous telangiectasia	14	18	cerebellar atrophy	350	3.68	0.51	1.53
FAT–2	female	10	4	ataxia, oculocutaneous telangiectasia, dysarthria	25	44	cerebellar atrophy	376	2.32	1.03	1.23
FAT–3	male	7	2	ataxia, oculocutaneous telangiectasia	16	22	cerebellar atrophy	183	5.36	1.16	1.77

Note: SARA: Scale for Assessment and Rating of Ataxia.

ICARS: International Co-operative Ataxia Rating Scale.

MRI: Magnetic Resonance Imaging.

^a^. Range of blood α-fetoprotein normal value: 0–13.6 ng/ml;

^b^. Range of blood IgG normal value: 7.23–16.85 g/L;

^c^. Range of blood IgA normal value: 0.69–3.82 g/l;

^d^. Range of blood IgM normal value: 0.63–2.77 g/l.

### Ethics statement

The study was approved by the Ethics Committee of Xiangya Hospital of Central South University in China (equivalent to an Institutional Review Board). This study was conducted according to the principles of the Declaration of Helsinki. Three affected individuals as well as their parents and 500 Chinese Han unaffected individuals as a healthy control were recruited in the study. Written informed consents were obtained from the 500 healthy controls and parents of the three probands to publish these case details because they did not have the capacity to understand and sign written informed consents.

### Targeted capture and next-generation sequencing

Genomic DNA was extracted from peripheral blood leukocytes by standard methods. The qualified genomic DNA sample was randomly fragmented by ultrasonoscope (Covaris S2, Massachusetts, USA) and the size of the library fragments was mainly distributed between 200 to 250bp. Next, purified DNA was treated with T4 DNA polymerase, T4 phosphonucleotide kinase and the Klenow fragment of *Escherichia coli* DNA polymerase to fill 5' overhangs and remove 3' overhangs. Terminal A residues were added following the incubation with dATP and the Klenow 3'-5' exo-enzyme by standard Illumina protocols. Then adapters were ligated to both ends of the resulting fragments.

A customized 2.1M Human capture array (Roche NimbleGen, Madison, WI) was designed to capture the fragments including exons, splice sites, and the adjacent intron sequences of the *ATM* gene, with subsequent sequencing performed with 90bp paired-end reads on a HiSeq2000 instrument (Illumina, San Diego, CA). Sequence reads were mapped to reference genomic DNA (UCSC hg19) with Burrows-Wheeler Alignment software for the subsequent variant analysis[9]. SNPs and indels were identified by using GATK software. Previously identified common variants (frequency > 1%) and synonymous substitutions were filtered out using public databases including dbSNP 142, HapMap samples, and the 1000 Genome Project (http://www.1000genomes.org). Potential disease-causing variants were evaluated using reference tools such as SIFT, Polyphen–2, as well as Mutation Taster predictions. All raw data available from the NIH Short Read Archive with the accession number SRP060492.

### PCR, RT-PCR and Sanger sequencing

PCR-based Sanger sequencing was performed to analyze the missense, nonsense, frameshift, and splicing mutations in the probands and their parents. Primer 5.0 was used to generate primers for the amplification of the target gene sites and related flanking sequences. The sequences were compared with the annotated *ATM* gene reference sequence (NM_000051) to confirm the candidate variants. Additionally, reverse transcription-PCR (RT-PCR) and Sanger sequencing were performed to verify the effect of c.1608-2A>G splicing mutation detected in proband of FAT–1 and his mother using the following primers: forward: 5-CACCTTCAGAAGTCACAGA ATGA–3’, reverse: 5’-GCCAATACTGGACTGGTGCT–3’.

### Cell Lines

Epstein–Barr virus-immortalized lymphoblastoid cell lines (LCLs) were established from the proband of FAT–3 (c.8110T>G, Hom) and normal control. They were grown in RPMI 1640 (Invitrogen, Carlsbad, CA) supplemented with 10% fetal bovine serum (FBS; Invitrogen) and cyclosporin A (0.2 mg/ml) at 37°Cwith 5% CO_2_.

### Western blot analysis

LCLs were lysed by RIPA buffer (50 mM Tris/pH 8.0, 150 mM NaCl, 1% NP–40, 2 mM EDTA, and protease inhibitor cocktail). Proteins extracts were separated on a 6% SDS-polyacrylamide gel and transferred to nitrocellulose membranes. The membranes were blocked with 5% nonfat milk and incubated with 1:1000 anti-ATM (Cell Signaling Technology, Boston, MA) and β-actin. The anti-rabbit or anti-mouse secondary antibodies coupled to horseradish peroxidase were detected using an ECL kit (Millipore, Bedford, MA). Quantification was performed using ImageJ software.

## Results

### Molecular analysis

**Fig 2:**
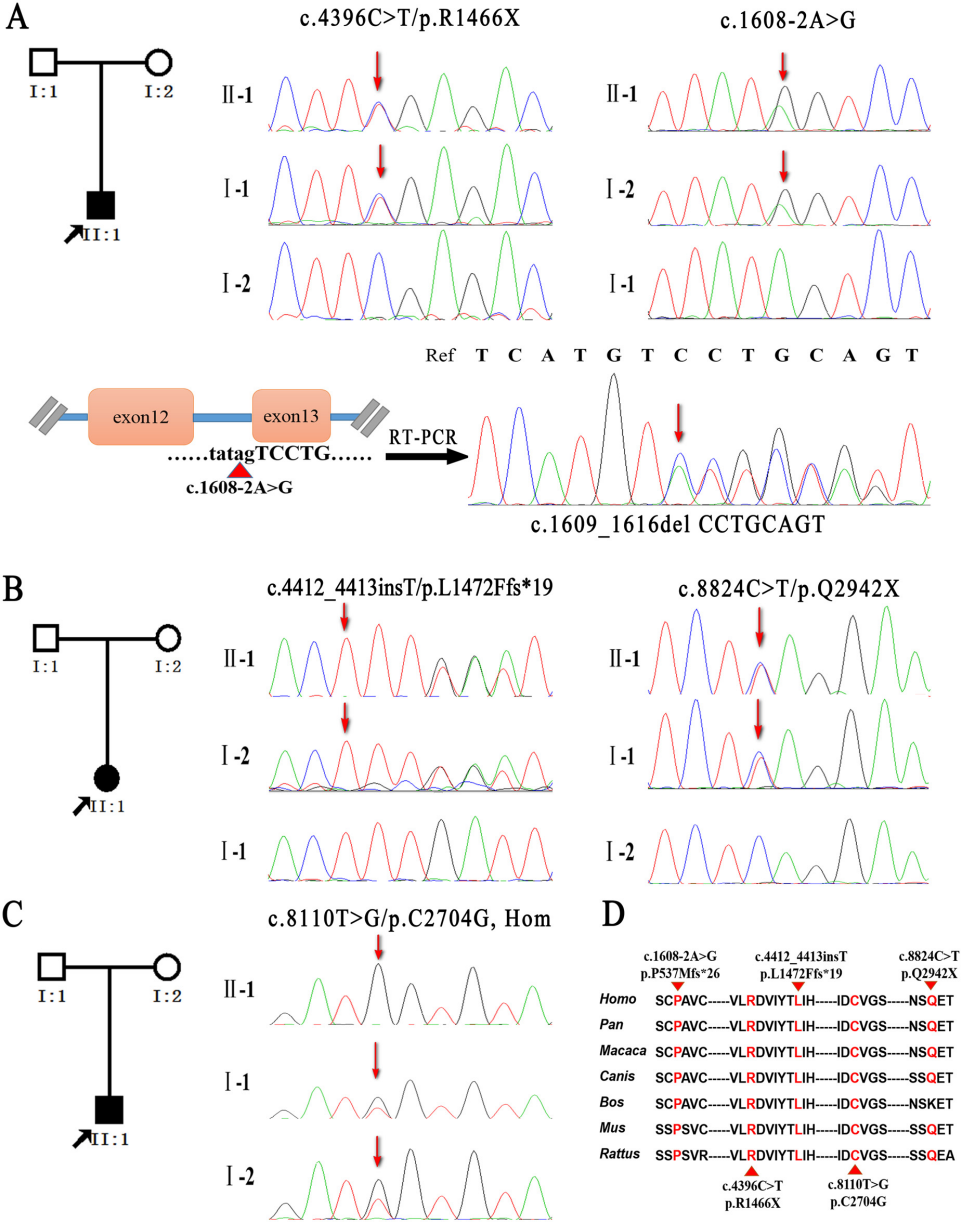
Pedigrees and putative pathogenic mutations. Segregation of mutations in FAT–1, FAT–2 and FAT–3 pedigrees was determined by Sanger sequencing (represented by arrows) (A-C). The splicing mutation of FAT–1 pedigree was verified via RT-PCR and Sanger sequencing (A). The conserved protein residues were targeted by the mutations identified in the patients (D).

Targeted next-generation sequencing for *ATM* gene was performed on each proband and generated an average of 18.25 million mapped reads of sequence with 226.33-fold average coverage and 99.3% sufficient coverage. We detected more than 12 thousand variants in each patient: 10981 SNPs and 1843 Indels in FAT–1 proband, 13222 SNPs and 2472 Indels in FAT–2 proband, 12426 SNPs and 2320 Indels in FAT–3 proband (Table 2). Initial variants were filtered out using public databases (dbSNP 142, HapMap samples, and the 1000 Genome Project) in combination with functional prediction. Using Sanger sequencing, we identified five disease-causing variants on *ATM* gene in three probands, including two pairs of heterozygous variants (FAT–1:c.4396C>T/p.R1466X, c.1608-2A>G; FAT–2:c.4412_4413insT/p.L1472Ffs*19, c.8824C>T/p.Q2942X) and one pair of homozygous variants (FAT–3: c.8110T>G/ p.C2704G, Hom) (Fig 2, Table 3, S1 Fig), which were segregated with their parents and absent in 500 unaffected healthy controls. Notably, except for one missense mutation (c.4396C>T) reported previously [10,11], four of these mutations were novel, including splicing, frameshift, nonsense and missense mutations. The novel frameshift (c.4412_4413insT) and nonsense mutations (c.8824C>T), as well as the missense mutation (c.8110T>G) were predicted to be damaging by SIFT, Polyphen–2, or MutationTaster and resulted in amino acid substitutions in conserved regions of the ATM protein. In addition, the splice site mutation (c.1608-2A>G) was validated by RT-PCR and Sanger sequencing, and resulted in a transcript variant with the deletion of CCTGCAGT in the *ATM* cDNA (c.1609_1616del CCTGCAGT), which was predicted as abnormal ATM protein with frameshift of 26 amino acids (p.P537Mfs*26).

**Table 2:** Overview of NGS data production.

Family samples	FAT–1	FAT–2	FAT–3
Raw reads (mapped to hg19)	13364918	19035012	22369564
Reads Length (bp)	90	90	90
Capture specificity (%)	66.3%	64.1%	65.9%
Bases mapped to genome (Mb)	1202.84	1713.15	2013.26
Average raw coverage (%)	99.34%	98.90%	99.52%
Effective exons coverage (%)	99.43%	99.00%	99.57%
Mean depth of targeted region (X)	168.10	231.64	280.05
Total number of variants	10981	13222	12426
Total number of indels	1843	2472	2320
Number of variants in SNP database	10298	12199	10987

**Table 3:** Summary of putative pathogenic mutations validated via Sanger sequencing or RT-PCR.

Family	Exon	Nucleotide variation	Type	Effect	Sift	Polyphen–2	Mutation Taster	Comment
FAT–1	exon31	c.4396C>T	nonsense	p.R1466X	NA	NA	disease causing	known
	IVS13	c.1608-2A>G	splicing	c.1609_1616del CCTGCAGT (p.P537Mfs*26)	NA	NA	disease causing	novel
FAT–2	exon31	c.4412_4413insT	frameshift	p.L1472Ffs*19	NA	NA	disease causing	novel
	exon63	c.8824C>T	nonsense	p.Q2942X	NA	NA	disease causing	novel
FAT–3	exon57	c.8110T>G, Hom	missense	p.C2704G	damaging (0.01)	damaging (1)	disease causing	novel

Note: the damaging predictions on nonsense mutations (c.4396C>T, c.8824C>T), splicing mutation (c.1608-2A>G) and frameshift mutation (c.4412_4413insT) are not applicable via Sift and Polyphen–2.

NA: not applicable.

### Functional investigation

Western blot analysis was performed on LCLs derive from the proband of FAT–3 and normal control to investigate the protein expression of the c.8110T>G/p.C2704G homozygous mutation on *ATM* gene. Compared to wild type, the ATM protein amount of p.C2704G homozygous mutant was significant decreased, suggesting that c.8110T>G variant on *ATM* gene might be deleterious (S2 Fig).

## Discussion

AT is a multisystem autosomal recessive disorder caused by mutation of *ATM* gene. To date, more than 780 mutations have been reported (http://www.hgmd.cf.ac.uk/ac/gene.php?gene=ATM), include missense, nonsense, splicing, small indels, large deletions, and duplications in the 66 exons of the *ATM* gene without apparent hotspots. Most of missense mutations responsible for AT often lead to ATM protein underexpression [12]. In this study, we made the accurate genetic diagnoses on three AT patients due to typical clinical symptoms combined with identification of five causative variants of *ATM* gene via targeted next-generation sequencing. The finding of novel mutations would broaden the genotypic spectrum of the *ATM* gene, which is beneficial for better understanding the relationship between the genotype and phenotype of AT.

Precision medicine is a group of new strategy that not only improves the prevention, diagnosis and treatment on common diseases and rare diseases, but also enhances the application of computational and bioinformatic tools on clinical medicine. With regards to precision medicine for rare genetic diseases, arriving at a precise diagnosis is essential. Considering our previous report of AT patients via laborious direct Sanger sequencing, such a costly evaluation would not facilitate the evolution of accurate diagnoses of rare Mendelian disorders [5,13]. Currently, this requirement of time and expense is not only a major challenge for clinicians to make a clinical diagnosis efficiently but also for researchers to develop prompt technical, statistical and bioinformatic innovations to guide clinical practice. As a promising tool for molecular detection, such disease-specific NGS assays should be developed to achieve timely and precise diagnosis. A high-throughput data-based strategy would propel the data mining of biomedical information including genomic, molecular and cellular parameters, which represent the “new frontier” for precision diagnosis and lay a firm foundation for precision medicine.

## Supporting Information

S1 Fig Details of coverage read of putative pathogenic mutations. IGV-browser revealed sufficient coverage read of each causative variant aligned to *ATM* gene found by targeted next-generation sequencing (A: c.4396C>T; B: c.1608-2A>G; C: c.4412_4413insT; D: c.8824C>T; E: c.8110T>G, Hom).
(TIF)

S2 Fig Western blot analysis of c.8110T>G variant of *ATM* gene. Western blot analysis showed reduced ATM protein amount in the FAT–3 proband compared to healthy control (A, B). The assay was repeated three times.
(TIF)
