# Supplementary figures and images for: Targeted Next-Generation Sequencing Revealed Novel Mutations in Chinese Ataxia Telangiectasia Patients: A Precision Medicine Perspective
Source: PLoS One. 2015 Oct 6;10(10):e0139738. doi: 10.1371/journal.pone.0139738 (PMC4595474; doi:10.1371/journal.pone.0139738)

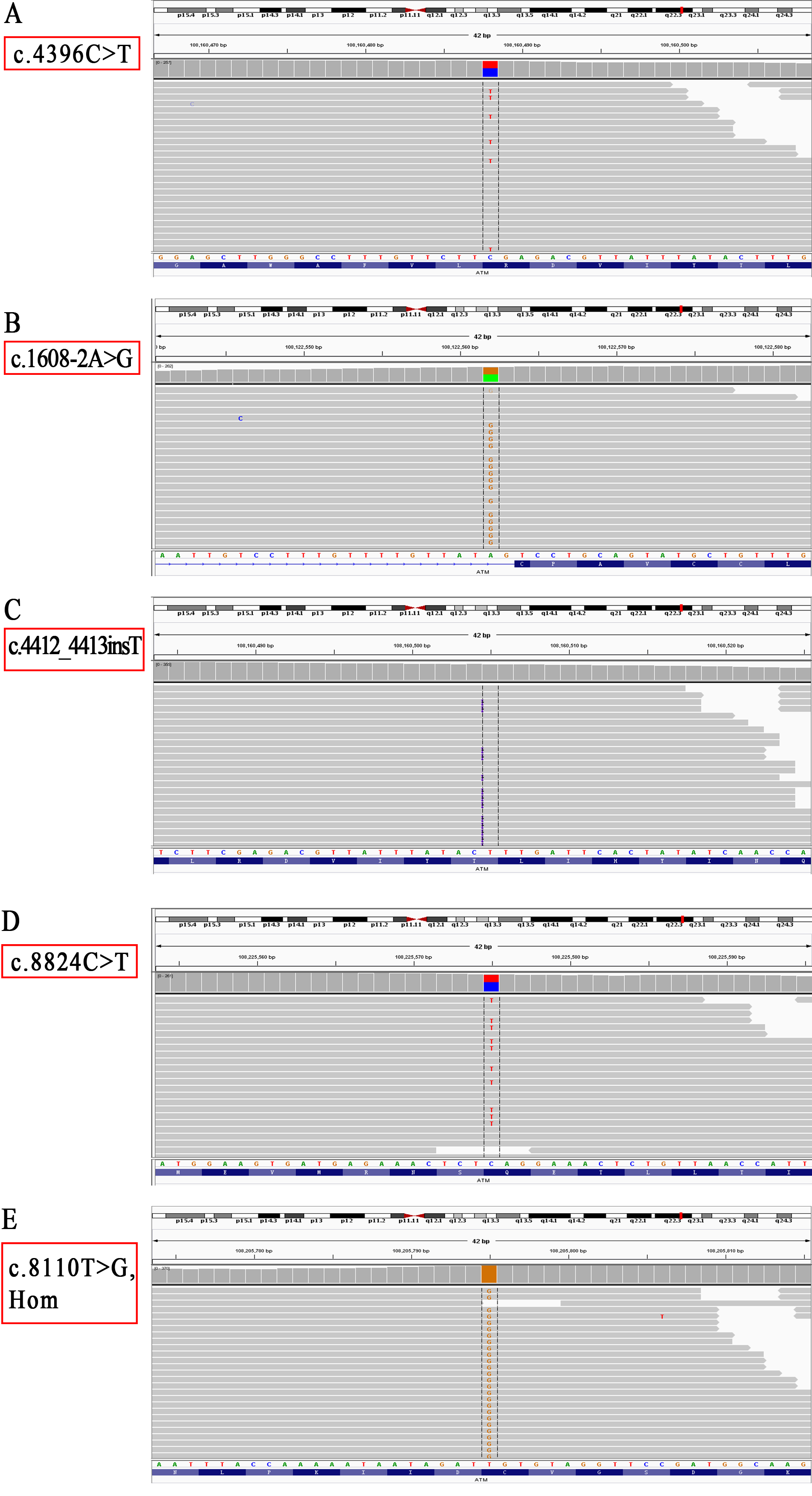

Supplement: S1 Fig — IGV-browser revealed sufficient coverage read of each causative variant aligned to ATM gene found by targeted next-generation sequencing (A: c.4396C>T; B: c.1608-2A>G; C: c.4412_4413insT; D: c.8824C>T; E: c.8110T>G, Hom). (TIF) [file pone.0139738.s001.tif]

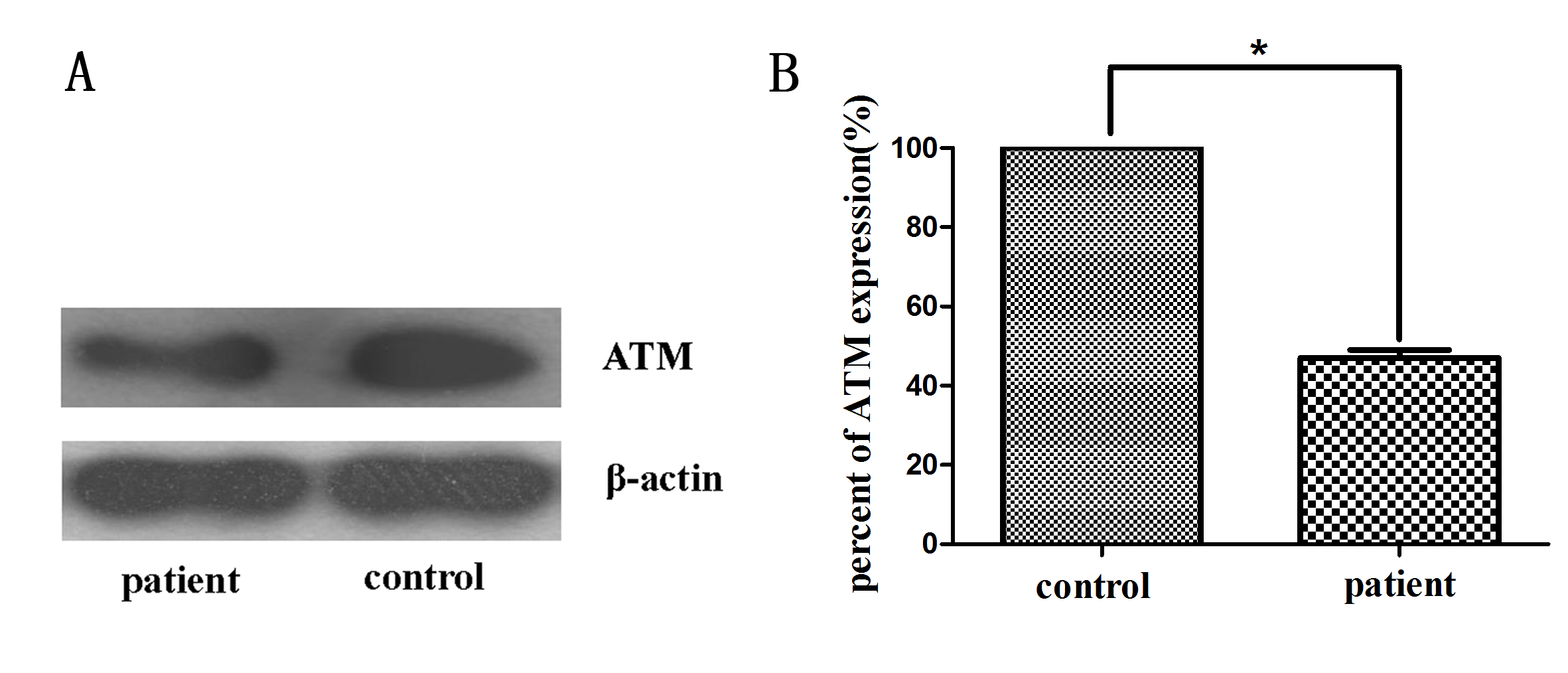

Supplement: S2 Fig — Western blot analysis showed reduced ATM protein amount in the FAT–3 proband compared to healthy control (A, B). The assay was repeated three times. (TIF) [file pone.0139738.s002.tif]
